# Systemic Characterization of the Pulp, Peels, and Seeds of the Yellow Puçá (*Mouriri grandiflora*) and Impacts of Simulated In Vitro Gastrointestinal Digestion on the Chemical, Biological, and Antioxidant Properties of Polyphenols

**DOI:** 10.1021/acsomega.5c02966

**Published:** 2025-06-20

**Authors:** Camila Mariane da Silva Soares, Rômulo Alves Morais, Maria Olivia dos Santos Oliveira, Romilda Ramos da Silva, Hermanny Matos Silva Sousa, Fabiana Queiroz, Glêndara Aparecida de Souza Martins

**Affiliations:** † Graduate Program in Food Science, Department of Food Science and Technology, 67739 Federal University of Lavras (UFLA), Lavras 37200-900, Brazil; ‡ Graduate Program in Food Science and Technology, Department of Food Science and Technology, 74385 Federal University of Tocantins (UFT), Palmas 77001-090, Brazil; § Kinetics and Process Modeling Laboratory, Department of Food Science and Technology, Federal University of Tocantins (UFT), Palmas 77001-090, Brazil

## Abstract

The yellow puçá (*Mouriri grandiflora*), a typical plant of the Cerrado, has nutritional and bioactive properties that are still little explored. The objective of this study was to evaluate the nutritional composition, physicochemical, and phytochemical properties, and to verify the effects of the in vitro gastrointestinal digestion simulation process on the phenolic profile, antioxidant potential, and in vitro biological properties of the pulp, peels, and seeds of the yellow puçá. The seeds presented high lipids, proteins, and fibers (17.35, 21.56, and 35.00 g 100 g^–1^, respectively). On the other hand, the pulp presented high contents of fructose (17.11 mg g^–1^) and malic acid (315.67 mg g^–1^). The peels presented the highest results for phytochemical analyses, amino acid profile, and mineral analysis. Regarding the phenolic profile, gallic acid was the compound marjoritary for pulp, peels, and seeds (652.01, 173.94, and 319.03 μg g^–1^, respectively). However, all phenolic compounds analyzed showed significant reductions when compared to the undigested sample and the intestinal phase (*p* < 0.05), indicating degradation processes or the formation of new compounds. Considerable decreases in the antioxidant potential and in vitro biological properties were observed. This is the first study to detail the complete characterization of all yellow puçá fractions.

## Graphical abstract

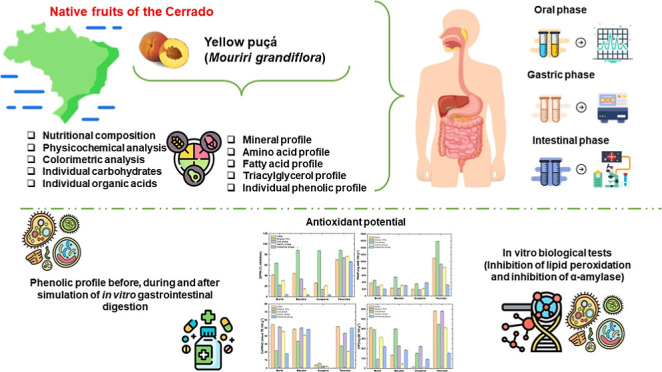

## 1 Introduction

The Brazilian Cerrado is widely recognized as a biodiversity hotspot due to its vast variety of species, many of which remain underexploited and underutilized. Among these, the fruits of the Cerrado stand out, presenting not only pleasant and unique sensory characteristics but also significant nutritional, functional, and therapeutic potential due to the presence of bioactive and antioxidant compounds, such as phenolics, vitamins, and carotenoids, among other compounds.^1,2^ Although some of these species are already used, primarily in a limited manner by local communities, being consumed in their natural state or processed into juices, liqueurs, ice creams, jams, and artisanal sweets, there is considerable exploration potential that has not yet been fully exploited.^3,4^ A relevant example of this wealth is the yellow puçá (*Mouriri grandiflora*), a species that has characteristics that are particularly favorable for a healthy and nutritious diet.

*M. grandiflora*, which belongs to the Melastomataceae family, is a fruit plant that produces edible fruits, popularly known as yellow puçá or camutim. Its fruits have a globose berry that contains a single seed surrounded by a fleshy orange pulp with a sweet flavor, and the remains of a calyx crown its yellow peel. These characteristics give the puçá a highly appreciated flavor.^5^ The medium-sized tree has elongated branches with small flowers ranging from white to pink along its woody branches.^6^ Rufino et al.^7^ highlighted the potential of puçá fruit as a valuable resource, owing to its significant levels of bioactive compounds, including vitamin C, carotenoids, and phenolic compounds. The synergistic effects of these components contribute to the fruit’s exceptional antioxidant capacity, positioning it as a promising candidate for sustainable agricultural practices and innovative product development. In addition to its considerable nutritional value, puçá has amazing potential for producing food and nutraceutical products, with potential benefits for preventing several diseases.^5^

Polyphenols present in native fruits have attracted increasing interest due to their therapeutic potential and health benefits, especially in preventing heart and neurological diseases.^8^ However, polyphenol absorption is inefficient, as most of these compounds are in their conjugated form and need to be hydrolyzed by intestinal enzymes or the microbiota of the digestive system to be absorbed. It is estimated that only 5–10% of ingested polyphenols are absorbed in the small intestine, while the majority are metabolized in the large intestine.^9,10^ Understanding the physicochemical transformations that foods undergo during digestion and the factors that impact nutrient bioaccessibility, bioavailability, and digestibility is crucial for developing functional foods. Bioaccessibility specifically refers to the proportion of a compound that is liberated from the food matrix and dissolved in the aqueous phase of the chyme, thereby becoming available for absorption across the intestinal membrane into the bloodstream.^11,12^ Bioavailability, on the other hand, describes the total amount of a compound released and absorbed, reaching the bloodstream, and distributed to different tissues throughout the body.^13^ Digestibility concerns explicitly the fraction of food components that transform potentially accessible material (present in soluble and insoluble digest fractions) through the physical and chemical processes that occur in the lumen.^11,14^

A more comprehensive understanding of these processes is essential for determining optimal daily nutrient intake recommendations and identifying processing conditions that enhance the health-promoting properties of bioactive compounds.^15^ Despite the growing scientific interest in native fruits of the Brazilian Cerrado, such as the yellow puçá (*M. grandiflora*), there are still important gaps in the literature regarding the complete characterization of its nutritional, functional, and bioactive properties, especially related to the digestibility, bioaccessibility and bioactivity of its compounds after the gastrointestinal digestion process. Most studies are limited to identifying the basic composition or consumption by local communities, neglecting crucial steps such as simulating in vitro digestion, which is essential to assess the real availability of bioactive compounds in the human body. Furthermore, to date, there are no studies in the literature that address the different parts of the fruit (pulp, peel, and seeds) in an integrated manner, disregarding its full potential for the development of sustainable functional foods and nutraceuticals. Therefore, this study stands out for using the standardized INFOGEST protocol to simulate gastrointestinal digestion in vitro, enabling a comprehensive and comparative evaluation of the physicochemical transformations, the release of bioactive compounds, and their antioxidant and biological properties in different fractions of the yellow puçá. This approach is unprecedented for this species and represents a significant advance in the understanding of the bioavailability and full use of underexploited native fruits, contributing to the valorization of the Cerrado biodiversity and the development of new products with functional and therapeutic appeal.

## 2 Materials and Methods

### 2.1 Chemical Reagents

The reagents neocuproine, thiobarbituric acid, potassium sulfate, sodium carbonate, sodium molybdate, ethanol, aluminum chloride, hydrochloric acid, 2,2-diphenyl-1-picrylhydrazyl (DPPH^•^), tripyridyl triazine (TPTZ), ferric chloride, ascorbic acid, potassium persulfate, potassium permanganate and α-amylase (30 U mg^–1^), pepsin (535 U mg^–1^), pancreatin (activity equivalent to 8× USP), and bile salts, such as sodium glycodeoxycholate (0.8 mM), sodium taurodeoxycholate hydra (0.45 mM), and sodium taurocholate hydrate (0.75 mM). Standards glucose, xylose, fructose, cellobiose, arabinose, citric acid, ascorbic acid, malic acid, and tartaric acid were used to analyze individual carbohydrates and organic acids. All other standards and reagents were purchased from Sigma-Aldrich (São Paulo, Brazil). All chemical reagents were of analytical grade.

### 2.2 Samples

The fruits of the yellow puçá (*M. grandiflora*) were collected in the Cerrado of the municipality of Lajeado, Tocantins, Brazil (Latitude—9°61′84″ S, Longitude—48°35′51″ W) between October and December 2022. The fruits were harvested manually and then subjected to cleaning, selection, and classification processes, with only ripe fruits in good condition being selected. The fruits were sanitized in chlorinated water at 100 mg L^–1^ for 15 min. After sanitization, the fruit fractions (pulp, peels, and seeds) were separated manually. After separation, the fruit fractions were dried in a circulation oven at 50 °C for 48 h. After drying, the fractions were ground in a commercial blender until they reached a standard particle size of 1.00 mm. They were stored in low-density polyethylene plastic bags at −20 °C until the analysis.

### 2.3 Nutritional and Physicochemical Composition

All analyses were determined according to the Association of Official Analytical Chemists.^16^ Methods 923.03 and 925.09, respectively, were used to determine ash and moisture content. The fat content was evaluated by the Soxhlet method, with *n*-hexane as the extracting solvent, during 6 h of reflux (method 920.85). The protein content was determined by the Kjeldahl method, using a conversion factor of 6.25 (method 920.87). The fibers were determined in a muffle furnace at 550 °C by gravimetric test (method 941.43). Carbohydrates were obtained by calculating the difference between the other constituents. The titratable acidity (947.05), pH (981.12), and soluble solids (932.12) were also analyzed. The total energy value was estimated considering the conversion factors of 4 kcal g^–1^ for proteins and carbohydrates and 9 kcal g^–1^ for lipids, and expressed in kcal 100 g^–1^ of the sample. Color analysis was performed at 25 °C using a digital colorimeter (Minolta CR_4000_, D_65_ light source in L*, a*, b*, chroma, and hue color space in the CIELAB system). All results were expressed as mean ± standard deviation (g 100 g^–1^). The extraction of starch and pectin from the pulp, peels, and seeds of the yellow puçá followed the protocols proposed by de Castro et al.^17^ with modifications made by Alves Morais et al.^18^ and Torralbo et al.^19^ respectively. The starch and pectin yields will be calculated using eq 1. The yields will be expressed in grams per hundred grams (g 100 g^–1^).

1 $$y=\frac{p}{B_{i}}\times 100$$

where *y* is the percentage yield of extracted starch or pectin, *p* (g) is the mass of extracted starch or pectin in grams, and *B*_i_ (g) is the initial mass of the sample.

### 2.4 Individual Carbohydrates and Organic Acids

For the quantification of individual carbohydrates and organic acids, the extracts were prepared as described by Alves Morais et al.,^20^ and the injection was made with 10 μL of each extract. Carbohydrates and organic acids were determined by high-performance liquid chromatography (HPLC) using an Agilent model 1260 Infinity II equipment equipped with a refractive index detector (RID) at 40 °C. The analysis was performed with a Supelcogel C_610H_ chromatographic column (30 cm × 7.8 mm) and a Supelguard C_610H_ precolumn (5 cm × 4.6 mm) from Sigma-Aldrich. The mobile phase consisted of a 0.1% phosphoric acid (H_3_PO_4_) solution in deionized water, with a flow rate of 0.5 mL min, total run time of 30 min, and oven temperature of 40 °C. Glucose, xylose, fructose, cellobiose, and arabinose were used as standards for the identification of carbohydrates, and citric, ascorbic, malic, and tartaric acids were used as standards for the identification of organic acids at a wavelength of 215 nm. The results were expressed in mg g^–1^.

### 2.5 Bioactive Compounds

#### 2.5.1 Recovery of the Extracts from Yellow Puçá Parts and Total Phenolic Content

To obtain extracts from the pulp, peels, and seeds of yellow puçá, 25 g of the samples were diluted in 100 mL of 80% (v/v) ethanol. Subsequently, the samples were subjected to sonication using an ultrasonic bath (EGS 5HD, 40 kHz, 300 W, Enge Solutions, São Paulo, Brazil) at 40 °C for 60 min. Afterward, the extracts were filtered (Whatman no. 541, 125 mm) and stored in amber vials at −20 °C for later analysis. These extracts were used to determine the phytochemical content of the respective fractions. Total phenolic contents were determined using the Folin–Ciocalteu spectrophotometric method according to Singleton and Rossi.^21^ For this purpose, 1 mL of the fruit fraction extracts was added to a solution composed of 0.2 mL of Folin–Ciocalteu reagent (10%), 2 mL of distilled water, and 1 mL of sodium carbonate (4%). The mixture was homogenized and kept in the dark for 2 h at room temperature. The absorbance was recorded at a wavelength of 750 nm, and the results were reported as milligrams of gallic acid equivalents per 100 g (mg GAE 100 g^–1^).

#### 2.5.2 Total Flavonoids and Anthocyanins

Flavonoid and anthocyanin content were determined using the method outlined by Francis and Markakis.^22^ In brief, 1 g of each dried sample was dissolved in 10 mL of an extraction solution consisting of 1.5 N HCl in 85% ethanol (v/v). The samples were homogenized, transferred to a 50 mL volumetric flask, and extracted for 13 h under refrigeration in an inert light environment. After extraction, the extracts were filtered using Whatman no. 1 filter paper. Absorbance was measured at 535 nm for anthocyanins and at 374 nm for flavonoids using a spectrophotometer. The results were expressed in mg per 100 g (mg 100 g^–1^).

#### 2.5.3 Total Carotenoids

Carotenoid extraction was conducted following the protocol described by Kimura and Rodriguez-Amaya.^23^ In brief, 1.0 g of each part of the yellow puçá fruit (pulp, peel, and seeds) was weighed and ground with 30 mL of acetone. This mixture was then transferred to a separatory funnel containing 50 mL of petroleum ether and filtered 3 times until the residue became colorless. The solution obtained was transferred to a separatory funnel containing 50 mL of petroleum ether and washed 3 times in sequence until the residue was colorless. Subsequently, 50 mL of acetone was added to the extract, followed by the controlled addition of 300 mL of distilled water. After separation of the phases, the lower fraction—composed predominantly of water and traces of solvents—was discarded. The remaining ethereal phase was then washed repeatedly (5 times) with distilled water to eliminate residues of water-soluble solvents. Then, the absorbance of the ethereal fraction was determined by spectrophotometry in the ultraviolet–visible (UV–vis) region. To quantify the carotenoids, the absorbance recorded at 450 nm was used, together with the molar extinction coefficient of β-carotene in petroleum ether (ε = 2592), as described in eq 2. The results were expressed as mean ± standard deviation, in micrograms of total carotenoids per gram of sample (μg β-carotene g^–1^).

2 $$Total\;carotenoids=\frac{A\times V\times 10^{4}}{E_{1cm}^{1\%}\times m}$$

where, *A* = extract absorbance at 450 nm; *V* = volume of the volumetric (mL); *m* = sample mass (g); *E*_1cm_^1%^ = extinction coefficient.

### 2.6 Mineral Profile

Minerals were determined using two digestion methods: nitro-perchloric, at a controlled temperature of 210 °C, and sulfuric, at a controlled temperature of 350 °C, according to the methodology described by Silva.^24^ First, 0.5 g of the sample was placed in a digestion tube, where 4 mL of concentrated solvent was added for both methods. Subsequently, the sample was taken to the digestion block for 2 h. After cooling, another 2 mL of nitric acid and 2 mL of concentrated hydrogen peroxide were added, and the sample was returned to the digestion block for another 2 h. Then, the volume of the samples was completed with ultrapure water to 25 mL in a volumetric flask, and then the samples were filtered through filter paper. Nitro-perchloric digestion was used to analyze the contents of magnesium, potassium, phosphorus, boron, sulfur, copper, manganese, zinc, and iron. The sulfuric digestion product was used to determine total nitrogen by micro-Kjeldahl distillation. Phosphorus was quantified with a molecular absorption spectrophotometer (model 700 Plus, FEMTO, São Paulo, Brazil), operating in the 660 nm range, while boron and sulfur were analyzed in the 420 nm range. Potassium was detected by flame photometry according to model 400 from Corning (New York, USA). Magnesium, zinc, copper, and iron contents were obtained with a flame atomic absorption spectrophotometer, model AAnalyst-400, from PerkinElmer (Waltham, USA). The results were expressed as mean ± standard deviation (g 100 g^–1^).

### 2.7 Amino Acids

Determining amino acids present in the pulp, peel, and seeds of yellow puçá was carried out following the protocol proposed by Hagen, Frost, and Augustin^25^ and White, Hart, and Fry.^26^ When weighing the sample and adding it to a hydrolysis tube, 9 mL of 6 N HCl with 3% w/v phenol was added, and the tube was aspirated, sealed, and placed in a thermal reaction block, leaving it for 24 h at 110 °C. Then, an aliquot of the internal standard AAAB was added and diluted to the tabulated volume. Following the dilution table, an aliquot was filtered and dried at 70 mTorr in a trap system with cryogenic nitrogen. It was neutralized with a solution of 0.2 N sodium acetate trihydrate, HPLC grade methanol, and 99% triethylamine (4:4:2 w/w/v) and dried again. At this step, PITC is added to derivatize the amino acids released by hydrolysis, forming PTC-amino acid. To the tube containing the derivatized amino acid crystals, 500 μL of diluent was added. Detection was performed at 254 nm by reversed-phase chromatography (30 μL injection loop, pH 6.40, in a binary linear gradient with a flow rate of 1 mL min^–1^ and column temperature of 58 °C), with eluent A being a buffer of 0.14 N sodium acetate, acetonitrile (240 mL 2000 mL^–1^ of 0.14 N sodium acetate), and triethylamine (1 mL 2000 mL^–1^ of 0.14 N sodium acetate). Eluent B was a 6:4 solution of acetonitrile (HPLC grade) and Milli-Q water (v/v). Quantification was performed using AAAB, aminobutyric acid, as an internal standard. Results are expressed as mean ± standard deviation (g 100 g^–1^).

### 2.8 Fatty Acid Profile and Triacylglycerol Composition

Lipids were extracted from the samples using the method (920.85) described by the Association of Official Analytical Chemists,^16^ followed by fatty acid esterification as described by Hartman and Lago.^27^ The fatty acid methyl esters (FAMEs) obtained were analyzed using gas chromatography coupled with flame ionization detection (GC-FID) on a Shimadzu GC 2010 chromatograph (Agilent Technologies Inc., Palo Alto, CA, USA), which was fitted with a flame ionization detector. Injection was performed in split mode (ratio 1:50) using a capillary column (SPTM-2560 Supelco, 100 m × 0.25 mm × 0.20 μm; Supelco Inc., Bellefonte, PA, USA). The temperature ramp involved heating the samples from 140 °C (for 5 min) to 240 °C (for 30 min) at a rate of 4 °C min^–1^, with the injector and detector set at 260 °C, and helium was used as the carrier gas. Fatty acids were identified and quantified by comparing the retention times of the sample peaks with those of the fatty acid standard mixture (Supelco_37_ Component FAME; Supelco, Bellefonte, PA, USA). The quantification was performed by normalization of the area and expressed as a percentage. The triacylglycerol profile of oils extracted from the pulp, peels, and seeds of yellow puçá was determined using the *PrOleos* software, as designed by Filho et al.^28^ This approach employs a random distribution model for fatty acids, assuming no positional preference between the *sn-1,3* and *sn*-*2* sites on the glycerol backbone, and relies on the fatty acid composition of the sample as the primary input for analysis.

### 2.9 Antioxidant Potential

#### 2.9.1 DPPH Scavenging Assay

The DPPH assay will follow the protocol established by Brand-Williams, Cuvelier, and Berset.^29^ Absorbance measurements will be recorded at a wavelength of 517 nm. The outcomes will be determined based on the radical scavenging activity and the extent of radical consumption in the reaction mixture. The results will be expressed as a percentage of inhibition, calculated using eq 3

3 $$\%\;inhibition=\left[1-\left(\frac{A_{A}}{A_{B}}\right)\right]\times 100$$

where *A*_A_ is the absorbance value of the sample, while *A*_B_ denotes the absorbance value of the control.

#### 2.9.2 Ferric Reducing Assay (FRAP)

The ferric reduction assay (FRAP) was determined according to Benzie and Strain.^30^ The FRAP solution was prepared by adding 2.5 mL of TPTZ solution (10 mmol L^–1^) diluted with HCl (40 mmol L^–1^), 2.5 mL of ferric chloride hexahydrate (20 mmol L^–1^), and 25 mL of sodium acetate buffer solution (pH 3.6). In a microplate, 20 μL of the obtained extracts and 280 μL of the FRAP reagent were added. The microplate was kept protected from light at 37 °C for 5 min. The absorbance of the solutions was measured at 593 nm. The reducing power of iron was determined from an ascorbic acid standard curve, and the results were expressed in mg of ascorbic acid equivalent per 100 g of sample (mg AAE 100 g^–1^).

#### 2.9.3 Total Reducing Capacity (TRC)

The total reducing capacity of hydrophilic and lipophilic compounds was evaluated according to the protocol proposed by Berker et al.^31^ In a centrifuge tube, 75 μL of the Folin–Ciocalteu reagent diluted in isobutanol 1:2 (v/v) was added to 50 μL of the sample. After 2 min, 875 μL of a 0.1 mol L^–1^ NaOH solution and 1.50 mL of ultrapure water were added to the mixture, which was then shaken for 10 s. The tubes remained at rest, protected from light, at room temperature, for 20 min. After the reaction, 250 μL of the solution was transferred to a microplate. The absorbance of the solutions was measured at 665 nm. The total reducing capacity was determined from a quercetin standard curve, and the results were expressed as mg of quercetin equivalent per 100 g of sample (mg QE 100 g^–1^).

#### 2.9.4 Copper Reducing Assay (CUPRAC)

The antioxidant activity evaluation based on the CUPRAC method was performed according to Apak et al.^32^ First, 50 μL of the obtained extracts were added to a test tube, followed by 1 mL of copper chloride (CuCl_2_), 1 mL of neocuproine (C_14_H_12_N_2_), and 1 mL of ammonium acetate (pH 7.0). Then, 250 μL of the solution was transferred to a microplate and kept at rest, protected from light, at room temperature, for 30 min. The absorbance of the solutions was measured at 450 nm. The reducing capacity of cupric ions was determined from a Trolox standard curve, and the results were expressed in mmol of Trolox equivalent per 100 g of sample (mmol TE 100 g^–1^).

### 2.10 In Vitro Biological Properties

#### 2.10.1 Inhibition of Lipid Peroxidation (ILP)

The inhibition of lipid peroxidation induced by Fe^2+^ in phospholipids, triacylglycerols, and proteins present in egg yolk will be assessed following the methodology outlined by Daker et al.^33^ and Margraf et al.,^34^ with slight modifications. The formation of peroxidation products will be monitored by measuring absorbance at 517 nm. A control assay (AC) will be simultaneously performed using ultrapure water instead of the extracts. The percentage of lipid peroxidation inhibition will be calculated using eq 4.

4 $$ILP=\frac{A_{c}-A_{a}}{A_{c}}\times 100$$

where ILP is the inhibition of lipid peroxidation (%); *A*_c_ is the absorbance of the control sample; *A*_a_ is the absorbance of the sample.

#### 2.10.2 α-Amylase Inhibition

The α-amylase inhibitory activity of the extracts was assessed following the method proposed by Ademiluyi and Oboh.^35^ In this procedure, 50 μL of the extract was combined with 500 μL of an α-amylase solution, prepared in a 20 mM sodium phosphate buffer (pH 6.9), and incubated at 25 °C for 10 min. Subsequently, 500 μL of a 1% starch solution was introduced into the mixture, and the incubation proceeded at 25 °C for an additional 10 min. The reaction was then triggered by adding 1 mL of a 60 nM 3,5-dinitrosalicylic acid (DNS) solution and then heating the mixture at 95 °C for 5 min. After the heating step, the samples were rapidly cooled in an ice bath, maintained at approximately 4 °C. To halt the reaction, 10 mL of distilled water was added, and the absorbance of the final solution was measured at 540 nm using a UV–vis spectrophotometer. A control sample was prepared by replacing the extract with 50 μL of 20 mM sodium phosphate buffer (pH 6.9), and α-amylase inhibition was calculated based on the method described in eq 5.

5 $$\alpha ‐Amylase\;inhibition=\frac{A_{c}-A_{a}}{A_{c}}\times 100$$

where *A*_c_ is the absorbance of the control sample, and *A*_a_ is the absorbance of the sample.

### 2.11 Simulation of In Vitro Gastrointestinal Digestion

The pulp, peel, and seed fractions of the yellow puçá were subjected to in vitro digestion simulation to verify the stability of the polyphenols during digestion. This step will be carried out based on the simulated digestion protocol proposed by INFOGEST.^11,12^ The method is divided into three phases: oral, gastric, and intestinal. For the oral phase of digestion, 0.25 g of dried samples were combined with 3.5 mL of simulated salivary fluid, 25 μL of CaCl_2_ solution, and 0.975 μL of distilled water. The pH of the resulting mixture was adjusted to 7.0 using a 0.1 M sodium hydroxide (NaOH) solution, then 0.5 mL of α-amylase (75 U mL^–1^) was added. The samples were then incubated at 37 °C under constant stirring at 100 rpm for 2 min. For the gastric phase, 7.5 mL of simulated gastric fluid, 20 μL of CaCl_2_ solution, and 0.700 μL of distilled water were added to the same reaction vessel used in the oral phase. The pH of the mixture was then adjusted to 3.0 with hydrochloric acid (HCl, 6 mol L^–1^), and 1.6 mL of pepsin (2000 U mL^–1^) was added. The samples were subsequently incubated in a thermostatically controlled water bath at 37 °C for 2 h. For the intestinal phase, 11 mL of simulated intestinal fluid, 40 μL of CaCl_2_ solution, 2.5 mL of bile salts, and 1.46 mL of NaOH (1 mol L^–1^) were introduced into the gastric phase reaction tubes. The pH was then neutralized to 7.0, adding 5 mL of pancreatin (800 U mL^–1^). The digestion process continued for 2 h at 37 °C under continuous stirring to mimic physiological conditions. After each digestion phase, enzymatic activity was stopped by placing the samples in an ice bath maintained at approximately 4 °C. Control experiments were conducted using identical proportions of samples and reagents, excluding digestive enzymes and bile salts. After each digestion phase, the resulting liquid was analyzed for antioxidant activity and phenolic compound profile, with comparisons made with samples before in vitro digestion. The method aims to evaluate digestion products and micronutrient release. As responses to the different phases of simulated digestion in vitro, the phenolic profile, antioxidant analyses (DPPH, FRAP, CUPRAC, CRT), and biological properties (inhibition of lipid peroxidation and inhibition of α-amylase) will be evaluated, as well as the calculation of bioaccessibility for the phenolic profile of the extracts.

### 2.12 Individual Phenolic Compounds

The phenolic composition of the pulp, peel, and seed extracts of yellow puçá was evaluated by high-performance liquid chromatography (HPLC) before and after each step of in vitro digestion (oral, gastric, and intestinal phases). Before injection, the extracts were filtered through a 0.22 μm membrane filter with a diameter of 13 mm. The identification and quantification of phenolic compounds were performed using an HPLC-DAD-UV–VIS system (AB Sciex LLC, Framingham, USA), equipped with a quaternary pump, degasser, injector, system controller, column oven, diode array detector, refractive index detector, and collector. The analysis was performed under the following conditions: column temperature maintained at 35 °C, Shim-pack VP-ODS analytical column (250 mm × 4.6 mm × 5 μm), and Shim-pack GVP-ODS guard column (10 mm × 4.6 mm × 5 μm). The mobile phase comprised two components: phase A, a 2% (v/v) aqueous solution of glacial acetic acid, and phase B, a mixture of methanol, water, and acetic acid in a 70:28:2% (v/v) ratio. The system functioned with a flow rate of 1.0 mL min^–1^, utilizing a gradient elution technique over a total runtime of 65 min. An injection volume of 20 μL was employed, and the phenolic compounds were detected at 280 nm. Calibration curves were obtained in duplicate, using ten different concentrations of each standard. The calibration curves were: trigonelline (*y* = 49881*x* – 553.26, *r* ^2^ = 0.9986), theobromine (*y* = 29533*x* – 47.97, *r* ^2^ = 0,9989), catechin (*y* = 11371*x* – 567.84, *r* ^2^ = 0.9993), vanillin (*y* = 69247*x* + 254.33, *r* ^2^ = 0.9986), and gallic acid (*y* = 3994*x* + 8047, *r* ^2^ = 0.9962), syringic acid (*y* = 49401*x* + 706.65, *r* ^2^ = 0.9990), chlorogenic acid (*y* = 29451*x* – 2224.70, *r* ^2^ = 0.9992), caffeic acid (*y* = 67928*x* – 2499.20, *r* ^2^ = 0.9991), *p*-coumaric (*y* = 100602*x* – 4097.70, *r* ^2^ = 0.9989), *o*-coumaric (*y* = 116320*x* – 6657, *r* ^2^ = 0.9990), *m*-coumaric acid (*y* = 130558*x* – 2496.20, *r* ^2^ = 0.9996), ferulic acid (*y* = 52609*x* – 5197.10, *r* ^2^ = 0.9997), rosmarinic acid (*y* = 30075*x* – 5006.10, *r* ^2^ = 0.9984), *trans*-cinnamic acid (*y* = 177527*x* – 8753, *r* ^2^ = 0.9989) and resveratrol (*y* = 79962*x* – 11,037, *r* ^2^ = 0.9983). The phenolic compounds were identified by comparing the sample peaks’ retention times with the standards’ retention times. The results were expressed in micrograms per gram (μg g^–1^).

### 2.13 Bioaccessibility Index

The bioaccessibility of phenolic compounds released from the pulp, peels, and seeds of yellow puçá during simulated gastrointestinal digestion and made available for absorption into the systemic circulation was assessed using the bioaccessibility index (eq 6).^36^

6 $$Bioaccessibility\;index\;\left(\%\right)=\frac{A}{B}\times 100$$

where *A* = total phenolic content after in vitro digestion and *B* = total phenolic content before in vitro digestion.

### 2.14 Statistical Analyses

Results are presented as mean ± standard deviation. Data were organized and processed in Excel spreadsheets. Significant differences were detected for the simulation of in vitro gastrointestinal digestion using the Tukey test or *t*-test to compare means at a significance level of 5% (*p* < 0.05) using Statistica 10.0 software (StatSoft Inc., Tulsa, OK, USA) when necessary. OriginPro 2022 software (OriginLab Corporation, Northampton, MA, USA) was used to analyze and plot the graphs.

## 3 Results and Discussion

### 3.1 Nutritional, Physicochemical, and Phytochemical Composition of the Pulp, Peels, and Seeds of the Yellow Puçá

Table 1 shows the results of the nutritional and physicochemical characterization of the pulp, peels, and seeds of the yellow puçá. As expected, the pulp and peels presented the highest moisture contents, with 68.26 and 66.61 g 100 g^–1^, respectively. High moisture content is directly related to the stability, quality, composition, and yield of the fruit. In this context, special care during harvesting, transportation, and processing of the yellow puçá is essential, as high moisture levels favor the development of deteriorating microorganisms, which can compromise the shelf life of the fruit.^18^ da Silva et al.^37^ observed higher moisture values in the black puçá (*Mouriri pusa*) pulp and peels, with 75.95 and 78.45 g 100 g^–1^, respectively. The observed differences can be attributed to factors such as harvest time, soil type, geographic variability, solar incidence, and variation among the analyzed species.

**Table 1:** Nutritional Composition, Carbohydrate and Organic Acid Profile, and Phytochemicals of the Pulp, Peels, and Seeds of the Yellow Puçá (*Mouriri grandiflora*)­^a^

Nutritional composition (g 100 g^–1^)	Pulp	Peel	Seeds
Moisture	68.26 ± 0.07	66.61 ± 0.02	53.96 ± 0.03
Ash	0.86 ± 0.01	0.98 ± 0.01	1.81 ± 0.01
Lipids	1.75 ± 0.11	2.56 ± 0.01	17.35 ± 0.04
Proteins	7.33 ± 0.20	7.45 ± 0.01	21.56 ± 0.01
Fibers	5.55 ± 0.09	9.03 ± 0.18	35.00 ± 2.90
Total carbohydrates*	21.8 ± 0.27	22.4 ± 0.18	5.33 ± 0.31
Energetic value (kcal 100 g^–1^)	132.27 ± 0.40	142.44 ± 0.19	263.71 ± 0.79
Starch	1.18 ± 0.10	1.17 ± 0.30	1.23 ± 0.50
Pectin	28.84 ± 3.00	28.26 ± 0.70	
Soluble solids	16.8 ± 0.17	16.7 ± 0.00	
pH	6.13 ± 0.25	5.81 ± 0.12	5.93 ± 0.01
Titratable acidity**	2.60 ± 0.20	3.80 ± 0.07	3.70 ± 0.61
L*	34.02 ± 1.27	26.10 ± 3.21	
°Hue (*h*)	71.80 ± 1.10	82.61 ± 0.83	
Chroma (C*)	43.58 ± 3.66	17.02 ± 0.80	
Carbohydrates (mg g^–1^)			
Sucrose	nd	nd	nd
Fructose	17.11 ± 0.23	3.47 ± 0.17	0.97 ± 0.02
Glucose	nd	nd	nd
Xylose	4.27 ± 0.10	2.91 ± 0.04	1.32 ± 0.01
Cellobiose	nd	nd	nd
Organic Acids (mg g^–1^)			
Ascorbic acid	55.96 ± 1.14	51.03 ± 0.24	68.41 ± 0.92
Tartaric acid	21.09 ± 0.33	111.48 ± 0.37	117.55 ± 0.40
Malic acid	315.67 ± 0.71	160.12 ± 0.08	nd
Citric acid	24.76 ± 1.48	88.11 ± 1.31	nd
Phytochemicals			
Total phenolics (mg GAE 100 g^–1^)	340.30 ± 1.20	840.96 ± 2.74	1010.57 ± 0.95
Carotenoids (μg β-carotene 100 g^–1^)	12.44 ± 0.35	17.40 ± 0.19	2.65 ± 0.10
Total anthocyanins (mg 100 g^–1^)	2.97 ± 0.12	6.43 ± 0.39	6.53 ± 0.92
Total flavonoids (mg 100 g^–1^)	35.76 ± 0.05	128.50 ± 0.071	160.00 ± 0.41

a Values expressed as mean ± standard deviation (*n* = 3); *carbohydrates calculated by difference; **values expressed in g citric acid 100 g^–1^; nd: not detected.; GAE: gallic acid equivalent.

Regarding lipids, the results indicate low levels in the pulp and peel of the yellow puçá, with 1.75 and 2.56 g 100 g^–1^, respectively, while the seeds presented a high content of 17.35 g 100 g^–1^ (Table 1). This lipid distribution pattern is typical in fruits since, in most plants, lipids are predominantly stored in the endosperm of the seeds due to genetic and metabolic factors that regulate the storage of energy reserves. da Silva et al.^37^ observed similar behavior for the black puçá, with low lipid levels in the pulp and peel and a high seed content. Regarding the protein content, it is essential to emphasize that, as defined by da Silva Sousa et al.,^38^ for a food to be considered a good source of protein, it must contain at least 12 g 100 g^–1^. Based on this criterion, the consumption of 100 g of pulp, peels, and seeds would represent 87.96%, 89.04%, and 100%, respectively, of the recommended daily protein intake for a healthy adult. These data highlight the nutritional potential of yellow puçá seeds, which, in addition to their high lipid content, also contribute significantly to the protein intake in the diet.

The results obtained for carbohydrates and energy values indicate differences in the content between the analyzed samples. The pulp and peels presented relatively similar carbohydrate contents, with 21.80 and 22.41 g 100 g^–1^, respectively, while the seeds had relatively lower levels (5.33 g 100 g^–1^). These carbohydrates are largely responsible for the energy value of the different fractions of the fruit. The pulp and peel have energy values of 132.27 and 142.44 kcal 100 g^–1^, respectively, while the seeds have a higher energy value (263.71 kcal 100 g^–1^), which can be attributed to the higher lipid content (17.35 g 100 g^–1^) in the seeds, compared to the pulp and peel (1.75 and 2.56 g 100 g^–1^, respectively). This higher lipid content in the seeds justifies their higher energy value since lipids provide a more concentrated amount of energy per gram compared to carbohydrates.^39^ Therefore, the distribution of lipids and carbohydrates among the different components directly reflects the caloric and nutritional value of each part of the fruit.

The results obtained for the pectin content in the pulp and peel of yellow puçá (28.84 and 28.26 g 100 g, respectively) indicate that these fractions present concentrations compatible with those of fruits rich in pectin, suggesting that they can be used as substitutes for commercial pectin. However, no positive results were found for the seeds of yellow puçá. Similar results were described by da Silva et al.^37^ when investigating black puçá, where the authors also observed high pectin contents in the pulp and peel but not in the seed. These values are consistent with those found in other tropical fruits, such as mango,^40^ and indicate that yellow puçá can be a promising source of pectin for food applications. The pH and titratable acidity results of the different fractions of yellow puçá (Table 1) indicate an expected correlation between these two variables. The pulp, with a pH of 6.13, presents the lowest titratable acidity (2.60 g citric acid 100 g^–1^), consistent with its greater alkalinity. On the other hand, the peels and seeds, with lower pH (5.81 and 5.93, respectively), have higher titratable acidity values (3.80 and 3.70 g citric acid 100 g^–1^, respectively). This pattern is commonly found in peels and seeds since there is an increase in the concentration of organic acids, such as citric and malic acid, which contribute to a more acidic pH and higher titratable acidity.^41^ These results on the distribution of acids in the different fractions of the fruit indicate that the pulp of the yellow puçá is more neutral, while the peels and seeds are more acidic, which may influence its use in food applications.

The results obtained for carbohydrates reveal a distinct distribution among the fruit fractions (Table 1). The pulp presented the highest concentrations of fructose and xylose (17.11 and 4.27 mg g^–1^, respectively), which is in line with the carbohydrate profile observed in many native fruits such as bacupari, tucumã, pupunha and yellow puçá, where reducing sugars, such as fructose, predominate due to their rapid conversion into energy sources.^37,38^ The absence of sucrose in the three fractions may suggest a distinct metabolic pathway for forming sugars in the yellow puçá, emphasizing fructose. Since, in more acidic environments, sucrose undergoes a hydrolysis process, where its molecule breaks down and is partially divided into the monosaccharide fructose and glucose.^42^ Compared to other traditional fruits, such as apple^43^ and mango,^44^ which have more balanced carbohydrate profiles between fructose and glucose, the yellow puçá stands out for its predominance of fructose in the pulp and a moderate xylose content in the skins and seeds, which may reflect differences in the specific metabolic characteristics of the plant.

Regarding organic acids, the acidity of yellow puçá is distributed differently among the fruit fractions, with emphasis on the pulp, which presented the highest levels of malic, ascorbic, and citric acid (315.67, 55.96 and 24.76 mg g^–1^, respectively) (Table 1). These compounds synergistically act as significant protectors on the myocardium act in ischemic lesions and, together with their salts, contribute to iron absorption.^45,46^ On the other hand, tartaric acid, found in higher concentrations in the peels and seeds (111.48 and 117.55 mg g^–1^, respectively), is a compound commonly associated with fruits with an acidic flavor and a potent antioxidant. The higher concentration of tartaric acid in the peels and seeds, compared to the pulp (21.09 mg g^–1^), may reflect a defense mechanism or a specific functional adaptation of these fractions about abiotic stresses.^47^ These findings on the composition of organic acids have direct implications for the methods and processes to be applied to the different fruit fractions, influencing their sensory and functional properties.

The results for phenolic and carotenoid compounds in yellow puçá indicate a significant distribution of these phytochemicals in the different fractions of the fruit (Table 1). The peel and seeds showed high concentrations of phenolic compounds (840.96 and 1010.57 mg GAE 100 g^–1^, respectively). The peels and seeds of many native tropical fruits, such as buriti and açaí, also demonstrate high levels of phenolics, exerting protective and antioxidant action.^48,49^ The pulp, on the other hand, showed a more moderate concentration of phenolics (340.30 mg GAE 100 g^–1^). da Silva et al.,^37^ when studying black puçá, reported a relatively lower number of phenolic compounds in the pulp (288.08 mg GAE 100 g^–1^) compared to the peels and seeds. Despite this, all fractions of the yellow puçá can be classified as good sources of phenolic compounds since, according to criteria established by Vasco et al.,^50^ fruits with levels higher than 500 mg GAE 100 g^–1^ are considered rich in this compound. Therefore, the presence of phenolic compounds is relevant, as they contribute significantly to foods’ antioxidant activity and exert potential therapeutic effects.^51^

The distribution of carotenoids and anthocyanins in the different fractions of yellow puçá suggests a phytochemical profile with nutritional and functional relevance (Table 1). Carotenoids are more concentrated in the peel (17.40 μg 100 g^–1^) compared to the pulp (12.44 μg 100 g^–1^), while the seeds present very low levels (2.65 μg 100 g^–1^). Carotenoids play crucial roles as precursors of vitamin A and antioxidants, contributing to eye health and physiological processes such as reducing blood glucose and reducing neurodegenerative diseases, in addition to favoring the body’s immune action.^52,53^ In contrast, anthocyanins, responsible for the color of several fruits, are more abundant in the peels (6.43 mg 100 g^–1^) and seeds (6.53 mg 100 g^–1^). Anthocyanins, with their antioxidant, anti-inflammatory, and potentially anticancer properties, add functional value to the yellow puçá, making its external fractions (peels) an important source of bioactive pigments.^54^

### 3.2 Mineral Profile

The mineral profile of the pulp, peels, and seeds of the yellow puçá presents significant differences in mineral concentrations (Table 2). The pulp presents satisfactory levels of nitrogen and potassium (8.06 and 7.00 g kg^–1^, respectively), demonstrating that it is rich in minerals essential for cell growth and muscle function. The recommended dietary intake of potassium for a healthy individual of both sexes is 4.70 g day^–1^.^55^ Therefore, the consumption of 100 g of both fractions of the yellow puçá would be enough to meet the entire daily demand. Therefore, potassium is crucial for electrolyte balance and blood pressure control, contributing to the prevention of cardiovascular diseases. However, a diet deficient in potassium can cause leg cramps, slow reflexes, muscle weakness, and irregular heartbeat, among other pathologies.^1^ On the other hand, it was observed that boron (7.13–13.80 mg kg^–1^) presented satisfactory concentrations among the different compounds. This mineral is fundamental to bone health and hormonal regulation and is a potent antioxidant.^56^

**Table 2:** Mineral Profile of the Pulp, Peel, and Seeds of Yellow Puçá (*Mouriri grandiflora*)­^a^

Minerals (g 100 g^–1^)	Pulp	Peel	Seeds
Nitrogen (g kg^–1^)	8.06 ± 0.20	9.63 ± 0.11	22.57 ± 0.28
Phosphorus (g kg^–1^)	0.54 ± 0.01	0.80 ± 0.05	1.44 ± 0.43
Potassium (g kg^–1^)	7.00 ± 0.33	9.00 ± 0.30	6.00 ± 0.71
Calcium (g kg^–1^)	0.80 ± 0.02	1.60 ± 0.48	0.70 ± 0.09
Magnesium (g kg^–1^)	0.90 ± 0.06	1.10 ± 0.05	1.00 ± 0.01
Sulfur (g kg^–1^)	0.15 ± 0.01	0.36 ± 0.01	0.96 ± 0.01
Boron (mg kg^–1^)	3.80 ± 0.19	5.03 ± 0.20	7.13 ± 0.29
Copper (mg kg^–1^)	0.28 ± 0.05	2.03 ± 0.74	1.41 ± 0.91
Iron (mg kg^–1^)	28.96 ± 0.20	34.31 ± 0.71	36.58 ± 0.57
Manganese (mg kg^–1^)	0.75 ± 0.05	1.37 ± 0.06	1.11 ± 0.09
Zinc (mg kg^–1^)	3.22 ± 0.03	4.76 ± 0.29	8.13 ± 0.58

a Values expressed as mean ± standard deviation (*n* = 2).

On the other hand, the peels have higher concentrations of copper (2.03 mg kg^–1^) and zinc (4.76 mg kg^–1^) (Table 2), which make them a good option for bone health and strengthening the immune system.^57^ In addition, they stand out for the significant amount of iron (34.31 mg kg^–1^), an essential mineral for the formation of hemoglobin and the transport of oxygen in the blood, which may be particularly beneficial in preventing anemia.^58^ The seeds of the yellow puçá, with high levels of iron (36.58 mg kg^–1^) and zinc (8.13 mg kg^–1^), have a highly nutritious mineral profile with notable benefits for immune function, wound healing, and maintenance of cellular integrity.^57^ Zinc, in particular, is an essential cofactor for several antioxidant enzymes and plays a crucial role in cellular metabolism and the immune response.^59^ Thus, each fraction of the yellow puçá contributes differently to promoting health, with concentrations of minerals that benefit everything from muscle function to strengthening the immune, bone, and neurological systems, and can be applied in various industrial sectors, such as cosmetics and pharmaceuticals.

### 3.3 Amino Acids

Analyzing essential and nonessential amino acids in different yellow puçá parts reveals significant nutritional value information (Table 3). Essential amino acids, which are indispensable for a series of metabolic functions and cannot be synthesized by the human body, are present in higher concentrations in the seeds, followed by the skin and pulp (3.09, 1.55, and 0.99 g 100 g^–1^, respectively). Among these amino acids, leucine and valine stand out in the seeds, with 0.73 and 0.40 g 100 g^–1^, respectively. This same behavior can be observed by da Silva et al.^37^ when studying the seed of the yellow puçá (*M. pusa*), where the authors reported higher concentrations of essential amino acids in the seed (3.45 g 100 g^–1^), with leucine and valine as the major amino acids (0.82 and 0.44 g 100 g^–1^, respectively). Leucine, in addition to being crucial for synthesizing muscle proteins, plays a central role in the process of muscle recovery and growth, contributes to controlling blood glucose levels, and helps regulate energy metabolism.^60^ Valine, which also participates in protein synthesis, is essential for maintaining the body’s nitrogen balance and immune system function.^61,62^ Both substances are indispensable, as they cannot be produced by the body and are required through food, especially in high-quality protein sources, such as yellow puçá seeds.

**Table 3:** Amino Acids from the Pulp, Peel, and Seeds of the Yellow Puçá (*Mouriri grandiflora*)­^a^

Amino acids (g 100 g^–1^)	Pulp	Peel	Seeds
Essentials			
Histidine	0.07 ± 0.01	0.11 ± 0.01	0.24 ± 0.03
Threonine	0.11 ± 0.02	0.17 ± 0.02	0.22 ± 0.05
Tyrosine	0.05 ± 0.01	0.10 ± 0.05	0.32 ± 0.02
Valina	0.16 ± 0.02	0.25 ± 0.00	0.40 ± 0.01
Methionine	0.01 ± 0.00	0.01 ± 0.00	0.06 ± 0.00
Isoleucine	0.12 ± 0.01	0.18 ± 0.07	0.30 ± 0.05
Leucine	0.21 ± 0.05	0.32 ± 0.06	0.73 ± 0.01
Phenylalanine	0.11 ± 0.02	0.18 ± 0.01	0.53 ± 0.05
Lysine	0.15 ± 0.02	0.23 ± 0.02	0.29 ± 0.06
Tryptophane	nd	<0.01	<0.01
∑ essentials	0.99 ± 0.05	1.55 ± 0.09	3.09 ± 0.28
Nonessential			
Glutamic acid	0.45 ± 0.05	0.53 ± 0.03	1.67 ± 0.01
Serina	0.26 ± 0.01	0.31 ± 0.01	0.32 ± 0.09
Glycine	0.16 ± 0.07	0.26 ± 0.01	0.72 ± 0.09
Taurine	<0.01	<0.01	<0.01
Arginine	0.18 ± 0.03	0.25 ± 0.02	1.84 ± 0.01
Alanine	0.24 ± 0.06	0.29 ± 0.10	0.36 ± 0.03
Proline	0.23 ± 0.01	0.30 ± 0.01	0.39 ± 0.05
Cystine	0.06 ± 0.00	0.05 ± 0.01	0.20 ± 0.02
Hydroxyproline	0.02 ± 0.00	0.03 ± 0.00	0.04 ± 0.01
∑ No essentials	1.60 ± 0.09	2.02 ± 0.11	5.54 ± 0.52
**∑ Total** **amino acids**	2.59 ± 0.08	3.57 ± 0.20	8.63 ± 0.74

a Values expressed as mean ± standard deviation (*n* = 2); nd: not detected; <0.01 = below the limit of quantification (LOQ < 0.01).

Regarding nonessential amino acids, which are synthesized by the body, the data indicate a similar pattern. The concentrations of nonessential amino acids in the peel and pulp follow a pattern similar to that observed for essential amino acids, with the seeds presenting the highest results (5.54 g 100 g^–1^), followed by the peel and pulp (2.02 and 1.60 g 100 g^–1^, respectively) (Table 3). The differentiation between the parts of the plant reinforces the importance of the seeds as a primary source of amino acids, both essential and nonessential, for the human diet. The main nonessential amino acids found in the seeds of the yellow puçá were glutamic acid, arginine, and glycine (1.67, 1.84, and 0.72 g 100 g^–1^, respectively). Thus, raw materials that present satisfactory concentrations of these amino acids act in metabolism and in maintaining health, in addition to being essential in the transmission of nerve signals to the brain, being one of the main excitatory neurotransmitters, in addition to acting in the cellular energy production cycle and the metabolism of ammonium, aiding in the detoxification of the organism, and acting in the production of nitric oxide, a compound that plays an essential role in the dilation of blood vessels and increasing circulation, contributing to cardiovascular health.^60,63,64^ Together, these amino acids not only perform essential metabolic functions but are also closely related to the proper functioning of the nervous system, cardiovascular health, and the energy balance of the organism. Thus, the amino acid profile of the different fractions of yellow puçá indicates its potential for nutritional and food applications, highlighting its importance in a balanced diet.

### 3.4 Fatty Acids

Table 4 shows that all oils analyzed presented higher concentrations of saturated fatty acids (93.34–98.35%) than monounsaturated (1.25–6.38%) and polyunsaturated (0.12–0.40%) fatty acids. These results indicate that the oils obtained from yellow puçá are rich in saturated lipids, comparable to other oilseeds native to Brazil, such as coconut oil (37.01–63.60%).^65^ Saturated fatty acids are widely used due to their stability and oxidation resistance, making these lipids ideal for formulating food products with greater shelf life, such as margarine and frying oils.^66^ The main saturated fatty acids present in the yellow puçá oils obtained from the pulp, peels, and seeds were palmitic (54.22–76.40%) and myristic (8.06–13.71%) acids (Table 4). The majority presence of these fatty acids gives the oil stability, increasing its resistance to oxidation and degradation at high temperatures, in addition to having antimicrobial and anti-inflammatory properties.^67^ However, the inclusion of oils rich in saturated fats should be evaluated with caution since uncontrolled consumption can negatively impact health, increase LDL cholesterol levels in the blood, and trigger inflammatory processes in the body, contributing to the development of chronic conditions, such as hypertension and type 2 diabetes.^68^

**Table 4:** Fatty Acid Profile of the Pulp, Peels, and Seeds of the Yellow Puçá (*Mouriri grandiflora*)­^a^

Fatty acids (%)	Pulp	Peel	seeds
Butyric (C4:0)	13.24 ± 0.85	nd	nd
Caproic (C6:0)	0.43 ± 0.03	0.63 ± 0.05	0.04 ± 0.00
Caprylic (C8:0)	nd	4.31 ± 0.32	nd
Capric (C10:0)	nd	0.57 ± 0.01	nd
Lauric (C12:0)	1.03 ± 0.02	1.92 ± 0.21	0.05 ± 0.01
Myristic (C14:0)	13.13 ± 1.87	13.71 ± 0.84	8.06 ± 0.21
Pentadecanoic (C15:0)	nd	9.18 ± 0.57	12.58 ± 1.32
Palmitic (C16:0)	55.10 ± 0.83	54.22 ± 1.58	76.40 ± 1.41
Heptadecanoic (C17:0)	10.24 ± 0.14	12.24 ± 0.92	nd
*cis*-10-heptadecanoic (C17:1)	nd	0.95 ± 0.06	nd
Stearic (C18:0)	0.17 ± 0.03	1.19 ± 0.12	0.25 ± 0.12
Oleic (C18:1)	6.38 ± 0.20	0.30 ± 0.06	2.48 ± 0.20
Linoleic (C18:2)	0.08 ± 0.01	0.37 ± 0.02	0.12 ± 0.05
Linolenic (C18:3)	0.20 ± 0.05	0.03 ± 0.01	nd
Arachidic (C20:0)	nd	0.22 ± 0.05	0.02 ± 0.00
Heneicosanoic (C21:0)	nd	0.16 ± 0.07	nd
Σ Saturated (SFA)	93.34 ± 0.47	98.35 ± 0.28	97.40 ± 0.81
Σ Monounsaturated (MUFA)	6.38 ± 0.20	1.25 ± 0.12	2.48 ± 0.33
Σ Poly unsaturated (PUFA)	0.28 ± 0.07	0.40 ± 0.05	0.12 ± 0.02

a Values expressed as mean ± standard deviation (*n* = 2); nd: not detected.

In addition to the nutritional implications, the fatty acids identified in the different oils from the parts of the yellow puçá can be applied in technological and industrial processes. Butyric acid, present in the pulp (13.24%), is particularly relevant for the cosmetics and pharmaceutical industries due to its antimicrobial and anti-inflammatory properties.^69^ Medium-chain fatty acids, such as caprylic (4.31%) and pentadecanoic (9.18%), present in the peel, have applications in the production of biodiesel and the food industry due to their efficiency in emulsification and potential antibacterial effect.^70^ Additionally, the high levels of oleic acid present in the pulp and seeds (6.38 and 2.48%, respectively) and stearic acid in the peels (1.19%) can be used for the formulation of healthy food products, such as vegetable oils and margarine, which benefit from the stability of these fatty acids at high temperatures.^68^ The lipid profile observed in yellow puçá, with a combination of saturated and unsaturated fatty acids, presents great potential for developing new ingredients in several industries, taking advantage of these compounds’ nutritional and technological characteristics.

### 3.5 Triacylglycerol Composition

The triacylglycerol (TAG) compositions of the oil samples are presented in Table 5, considering only fatty acids >0.5% of the total peak area. The main triacylglycerols in the pulp oil were BuPP, PMP, PPP, and HPP, representing approximately 54% of the triacylglycerol composition. On the other hand, the main TAGs found in the oil obtained from the peels were PMP, PPeP, PPP, and PHP, representing approximately 55% of the total triacylglycerols. The seeds, however, presented the TAGs MPPe, MPP, PePP, and PPP as major compounds, the sum of these four composing approximately 87% of the TAGS of the seed oil of the yellow puçá. The composition of the oils of the different fractions of the yellow puçá was predominantly composed of diunsaturated TAGs (PMP and HPP), followed by triunsaturated (PPP). These results corroborate the fatty acid composition shown in Table 4, demonstrating that it is rich in saturated fatty acids such as palmitic, myristic, and heptadecanoic acid. This same behavior can be observed by Alves Morais et al.^18^ when studying the TAGS profile in the different fractions of the buritirana fruit (*Mauritiella armata*), and found that the oils were composed of diunsaturated TAGs (POO and PLO), followed by monounsaturated (POP). Consequently, the predominant triacylglycerol (TAG) identified across all oil samples was PPP, with concentrations of 18.12%, 18.58%, and 45.53% in oils derived from the pulp, peels, and seeds, respectively. These findings highlight a clear correlation between the variations in the TAG composition of the oils and the distinct fatty acid profiles of each sample, underscoring how the lipid composition in different plant fractions influences the overall lipid structure and properties of the oils.

**Table 5:** Triacylglycerol Composition of Oils from Pulp, Peel, and Seed Oils of Puçá (*Mouriri grandiflora*)­^a^

Triacylglycerol (%)				
ECN	Acronym	Pulp	Peel	Seeds
22:0	BuMBu	0.74 ± 0.02		
24:0	BuPBu	3.12 ± 0.24		
25:0	BuHBu	0.57 ± 0.06		
32:0	BuMM	0.74 ± 0.09		
34:0	BuMP	6.20 ± 0.52		
35:0	BuMH	1.15 ± 0.02		
36:0	BuPP	13.02 ± 1.30		
36:1	BuOM	0.72 ± 0.02		
37:0	BuPH	4.85 ± 0.09		
38:0	CapPP		0.62 ± 0.01	
38:0	CMP		2.24 ± 0.72	
38:1	BuOP	3.02 ± 0.28		
39:0	CPeP		1.50 ± 0.10	
39:1	BuOH	0.56 ± 0.07		
40:0	CPP		4.42 ± 0.86	
41:0	CHP		2.00 ± 0.33	
42:0	CaPP		0.61 ± 0.01	
42:0	PLaM		0.99 ± 0.04	
43:0	PLaPe		0.66 ± 0.03	
43:0	PeMM		0.60 ± 0.01	
44:0	LaPP	0.98 ± 0.09		
44:0	PMM	3.07 ± 0.32	3.56 ± 0.44	
44:0	PLaP		1.95 ± 0.46	
44:0	MPM			1.54 ± 0.01
45:0	HMM	0.57 ± 0.08		
45:0	PMPe		4.78 ± 0.05	
45:0	PHLa		0.88 ± 0.02	
45:0	MHM		0.80 ± 0.06	
45:0	MPPe			4.78 ± 0.11
46:0	PMP	12.93 ± 1.02	14.09 ± 0.63	
46:0	PPePe		1.60 ± 0.05	
46:0	PeHM		1.08 ± 0.05	
46:0	MPP			14.49 ± 0.59
46:0	PePPe			3.71 ± 0.20
47:0	HMP	4.78 ± 0.62		
47:0	PPeP		9.46 ± 0.96	
47:0	PHM		6.34 ± 0.57	
47:0	PePP			22.52 ± 1.51
48:0	PPP	18.12 ± 0.94	18.58 ± 1.63	45.53 ± 1.20
48:0	SMP		0.62 ± 0.06	
48:0	PHPe		4.25 ± 0.22	
48:0	MHH		0.71 ± 0.05	
48:1	POM	3.00 ± 0.54		
48:1	MOP			
49:0	HPP	10.07 ± 0.36		
49:0	PHP		12.55 ± 0.92	
49:1	HOM	0.55 ± 0.07		
49:1	PHepP		1.02 ± 0.09	
49:1	PeOP			1.47 ± 0.11
50:0	HPH	1.85 ± 0.04		
50:0	SPP		1.23 ± 0.02	0.53 ± 0.02
50:0	PHH		2.82 ± 0.09	
50:1	POP	6.30 ± 0.55		4.47 ± 0.33
51:1	HOP	2.33 ± 0.41		
52:2	POO	0.73 ± 0.03		

a Results are presented as mean ± standard deviation (*n* = 2); ECN, equivalent carbon number and double bounds; Bu, butyric acid; M, myristic acid; P, palmitic acid; H, heptadecenoic acid; O, oleic acid; La, lauric acid; S, stearic acid; Pe, pentadecanoic acid; Hep, *cis*-10-heptadecanoic acid; Ca, capric acid; C, caprylic acid; Bu, butyric acid; Cap, caproic acid.

### 3.6 In Vitro Gastrointestinal Digestion and Bioaccessibility of Individual Phenolic Compounds

The phenolic profile of the pulp, peels, and seeds of yellow puçá before and after the in vitro digestion process is presented in Tables 6, 7, and 8, respectively. It is crucial to understand the behavior of the compounds throughout the digestive process, as their bioaccessibility is essential for potential health benefits, requiring concentrations available for intestinal absorption after digestion.^71^ Statistical analysis indicated significant differences between all individual phenolics, evidencing considerable variations throughout the stages of the digestive process (*p* < 0.05). Among the compounds identified in the extracts before in vitro digestion, gallic acid was the major compound for the pulp, peels, and seeds (652.01, 173.94, and 319.03 μg g^–1^, respectively). These results are highly satisfactory since fruits rich in gallic acids, such as yellow puçá, offer several health benefits, including antioxidant, anti-inflammatory, and antimicrobial properties, in addition to contributing to cellular protection against oxidative damage, strengthening the immune system and preventing cardiovascular diseases.^72–74^

**Table 6:** Bioaccessibility and Simulation of In Vitro Gastrointestinal Digestion of Yellow Puçá Pulp^a^

Individual phenolics (μg g^–1^)	RT	Nondigested ethanol 70%	Oral phase	Gastric phase	Intestinal phase	Bioaccessibility (%)
Trigonelline	3.49	1.42 ± 0.02^b^	0.93 ± 0.01^c^	1.96 ± 0.09^a^	0.25 ± 0.02^d^	17.60
Gallic acid	6.65	652.01 ± 0.96^a^	60.65 ± 0.20^d^	280.71 ± 0.87^b^	200.64 ± 0.02^c^	30.77
Theobromine	9.30	0.34 ± 0.10^b^	0.32 ± 0.08^b^	0.68 ± 0.05^a^	0.24 ± 0.01^b^	70.58
Catechin	10.49	0.80 ± 0.14^b^	0.33 ± 0.05^c^	1.92 ± 0.10^a^	0.73 ± 0.06^b^	91.25
Chlorogenic acid	12.19	0.62 ± 0.09^a^	nd	nd	0.30 ± 0.01^b^	48.38
Caffeic acid	14.38	1.19 ± 0.05^b^	0.89 ± 0.05^c^	4.89 ± 0.09^a^	0.76 ± 0.07^c^	63.86
Syringic acid	15.39	nd	nd	nd	nd	
Vanillin	16.80	nd	nd	nd	nd	
*p*-coumaric acid	20.65	1.52 ± 0.03^b^	0.37 ± 0.09^c^	4.24 ± 0.67^a^	0.10 ± 0.01^d^	6.57
Ferulic acid	23.57	0.24 ± 0.05	nd	nd	nd	
*m*-coumaric acid	25.95	0.31 ± 0.05^b^	0.17 ± 0.07^c^	2.17 ± 0.11^a^	nd	
*o*-coumaric acid	32.35	1.14 ± 0.01	nd	nd	nd	
Resveratrol	36.73	nd	nd	nd	nd	
Rosmarinic acid	42.83	13.30 ± 0.28	nd	nd	nd	
*trans*-cinnamic acid	50.43	3.32 ± 0.14^b^	1.04 ± 0.04^c^	11.16 ± 0.29^a^	0.77 ± 0.05^d^	23.19

a Results are presented as mean ± standard deviation (*n* = 2); means followed by the same lowercase letter in the same row do not differ statistically using the Tukey or *t*-test at 5% probability (*p* ≤ 0.05); nd: not detected; RT: retention time in minutes.

**Table 7:** Bioaccessibility and Simulation of In Vitro Gastrointestinal Digestion of Yellow Puçá Peel^a^

Individual phenolics (μg g^–1^)	RT	Nondigested ethanol 70%	Oral phase	Gastric phase	Intestinal phase	Bioaccessibility (%)
Trigonelline	3.49	3.34 ± 0.20^a^	1.33 ± 0.11^b^	0.22 ± 0.09^d^	0.99 ± 0.05^c^	29.64
Gallic acid	6.65	173.94 ± 0.58^b^	107.25 ± 0.85^d^	228.12 ± 0.63^a^	161.97 ± 0.74^c^	93.11
Theobromine	9.30	nd	0.70 ± 0.01^b^	9.23 ± 0.22^a^	0.55 ± 0.06^c^	
Catechin	10.49	3.42 ± 0.37^a^	0.87 ± 0.20^b^	nd	nd	
Chlorogenic acid	12.19	0.46 ± 0.06^b^	0.44 ± 0.01^b^	1.68 ± 0.10^a^	nd	
Caffeic acid	14.38	8.66 ± 0.39^a^	0.93 ± 0.08^b^	0.32 ± 0.01^c^	0.18 ± 0.02^c^	2.07
Syringic acid	15.39	1.61 ± 0.05^a^	0.07 ± 0.02^b^	nd	nd	
Vanillin	16.80	nd	0.09 ± 0.01^b^	0.34 ± 0.02^c^	nd	
*p*-coumaric acid	20.65	0.14 ± 0.07^b^	1.74 ± 0.01^a^	0.05 ± 0.03^c^	0.09 ± 0.01^c^	64.28
Ferulic acid	23.57	nd	0.35 ± 0.02^a^	0.25 ± 0.01^b^	nd	
*m*-coumaric acid	25.95	3.69 ± 0.06^a^	0.16 ± 0.04^b^	0.11 ± 0.04^b^	nd	
*o*-coumaric acid	32.35	0.12 ± 0.00^b^	0.28 ± 0.05^a^	0.24 ± 0.06^a^	nd	
Resveratrol	36.73	1.45 ± 0.05^a^	0.43 ± 0.02^c^	1.08 ± 0.10^b^	0.19 ± 0.01^d^	13.10
Rosmarinic acid	42.83	2.30 ± 0.58	nd	nd	nd	
*trans*-cinnamic acid	50.43	0.34 ± 0.01^b^	0.66 ± 0.06^a^	0.15 ± 0.02^c^	0.10 ± 0.01^c^	29.41

a Results are presented as mean ± standard deviation (*n* = 2); means followed by the same lowercase letter in the same row do not differ statistically using the Tukey or *t*-test at 5% probability (*p* ≤ 0.05); nd: not detected; RT: retention time in minutes.

**Table 8:** Bioaccessibility and Simulation of In Vitro Gastrointestinal Digestion of Yellow Puçá Seeds^a^

Individual phenolics (μg g^–1^)	RT	Nondigested ethanol 70%	Oral phase	Gastric phase	Intestinal phase	Bioaccessibility (%)
Trigonelline	3.49	nd	0.05 ± 0.01^c^	0.27 ± 0.02^b^	0.38 ± 0.02^a^	
Gallic acid	6.65	319.03 ± 0.40^a^	nd	84.58 ± 0.56^c^	80.30 ± 0.48^b^	25.17
Theobromine	9.30	4.65 ± 0.09^b^	0.10 ± 0.01^d^	17.11 ± 0.28^a^	1.58 ± 0.63^c^	33.97
Catechin	10.49	4.15 ± 0.20^a^	nd	0.74 ± 0.06^b^	4.02 ± 0.85^a^	96.86
Chlorogenic acid	12.19	1.24 ± 0.05^b^	0.35 ± 0.01^d^	1.77 ± 0.02^a^	0.76 ± 0.02^c^	61.30
Caffeic acid	14.38	0.62 ± 0.01^a^	0.10 ± 0.01^c^	0.10 ± 0.01^c^	0.46 ± 0.00^b^	74.20
Syringic acid	15.39	nd	nd	0.01 ± 0.00^a^	0.03 ± 0.00^a^	
Vanillin	16.80	nd	nd	nd	0.06 ± 0.00	
*p*-coumaric acid	20.65	0.61 ± 0.02^a^	0.03 ± 0.02^c^	nd	0.14 ± 0.01^b^	22.95
Ferulic acid	23.57	nd	0.14 ± 0.02^a^	0.17 ± 0.03^a^	0.19 ± 0.02^a^	
*m*-coumaric acid	25.95	0.43 ± 0.04^a^	nd	0.05 ± 0.00^b^	0.06 ± 0.00^b^	13.95
*o*-coumaric acid	32.35	nd	nd	0.19 ± 0.03^a^	0.23 ± 0.02^a^	
Resveratrol	36.73	nd	nd	nd	nd	
Rosmarinic acid	42.83	nd	nd	nd	nd	
*trans*-cinnamic acid	50.43	0.08 ± 0.01^b^	nd	0.15 ± 0.01^a^	nd	

a Results are presented as mean ± standard deviation (*n* = 2); means followed by the same lowercase letter in the same row do not differ statistically using the Tukey or *t*-test at 5% probability (*p* ≤ 0.05); nd: not detected; RT: retention time in minutes.

The analysis of the phenolic profile of the pulp revealed the presence of 12 individual phenolic compounds, of which only eight were bioaccessible at the end of the digestion process. The peels presented 12 compounds before digestion; only six remained bioaccessible in the intestinal phase. On the other hand, the seeds presented a more balanced behavior, with eight phenolic compounds quantified before digestion and 7 maintaining their bioaccessibility after the completion of the reactions (Tables 6–8). In all samples, a significant trend (*p* < 0.05) of reduction in phenolic compounds was observed throughout the in vitro gastrointestinal simulation. This pattern is consistent with previous studies that highlight the influence of enzymes and pH during digestion, resulting in phenolic compounds’ degradation, modification, and transformation.^75^ It is worth noting that the concentrations of phenolic compounds presented in Tables 6–8 correspond to values normalized to the volume of the initial sample, considering the dilutions caused by the addition of oral, gastric, and intestinal fluids during the simulation. This normalization ensures an accurate bioaccessibility comparison between the different digestive process stages.^71^

When evaluating the bioaccessibility of the compounds, it is noted that catechin was the phenolic that presented the greatest bioaccessibility for pulp and seed (91.25 and 96.86%, respectively). On the other hand, gallic acid was the most bioaccessible compound in the yellow puçá peel (93.11%). These values reflect high bioaccessibility, suggesting that these compounds’ absorption and utilization in the gastrointestinal tract are maximized. This behavior can be attributed to the interaction of phenolic compounds with food components, which can facilitate their release and absorption in the intestine. Recent studies indicate that gallic acid and catechin are widely found in various foods, and their availability for absorption by the body can be facilitated due to the formation of complexes with other substances present in the food.^36,76–78^

This same behavior was observed by Morais et al.^36^ when investigating the profile of phenolic compounds present in the pulp, peels and seeds of buritirana (*M. armata*). They identified significant variations between the respective phases of the in vitro gastrointestinal digestion simulation process and identified significant variations in the bioavailability and stability of phenolic compounds throughout the different digestive phases (oral, gastric and intestinal). These results show that each phase of the digestive process affects the different food matrices in a distinct and specific manner, reflecting both the release and the transformation of the bioactive compounds present. Such variations can be attributed to the physicochemical characteristics of the matrices—such as the composition of dietary fiber, the lipid and protein content, and the interaction with other constituents of the plant matrix—which modulate the solubilization and release of phenolic compounds.^11^ In addition, the food matrix plays a crucial role in the protection and/or degradation of phenolic compounds during digestion. For example, flavonoids and phenolic acids can undergo structural transformations, such as hydrolysis or conjugation, which affect their antioxidant activity and intestinal absorption.^73–76^

It can be concluded that the bioaccessibility of phenolic compounds in the pulp, peels, and seeds of yellow puçá varies significantly during the simulated in vitro gastrointestinal digestion process. While the pulp showed remarkable bioaccessibility for catechin (91.25%) and a considerable presence of gallic acid (30.77%) in the intestinal phase, the peels demonstrated the highest bioaccessibility for gallic acid (93.11%) among all compounds. The seeds exhibited remarkable bioaccessibility of catechin (96.86%), suggesting that this compound is highly available for absorption, while gallic acid had a more limited bioaccessibility (25.17%). The results obtained indicate that the bioavailability of phenolic compounds present in yellow puçá is significantly modulated by intrinsic characteristics of its chemical structure, as well as by intermolecular interactions with other constituents of the food matrix during the different phases of the digestive process.^11,12,36^ The substantial variation observed in the bioaccessibility of these compounds throughout simulated in vitro gastrointestinal digestion can be attributed to the chemical stability of phenolics under different physiological conditions (pH, enzymatic activity, presence of bile salts), as well as to their ability to form complexes with macronutrients. Phenolic compounds can undergo degradation, isomerization, or enzymatic conjugation reactions during gastric and intestinal digestion, directly affecting their solubility and subsequent absorption.^10–12^

Another relevant factor is the potential for enzymatic metabolism by digestive enzymes, such as esterases and glycosidases, which can modify the structure of phenolics, transforming them into metabolites of lower or higher polarity, which, in turn, influences their passive diffusion or active transport through intestinal cells.^75^ Therefore, understanding the mechanisms that regulate the release and transformation of phenolic compounds during digestion requires an integrated approach that considers both structural aspects of phenolics and the dynamic parameters of the food matrix. Overall, the presented data provide valuable insights into the digestive fate and bioaccessibility of phenolic compounds in yellow puçá, supporting its potential as a functional food ingredient.

### 3.7 Antioxidant Potential

The antioxidant potential of the pulp, peels, and seeds of yellow puçá was evaluated before and after the simulation of in vitro gastrointestinal digestion using the DPPH, FRAP, CUPRAC, and TRC assays, as shown in Figure 1. In general, a substantial reduction in the antioxidant activity of the different components of yellow puçá was observed during the digestive process, compared to the undigested samples. For the DPPH assay, the peels and seeds showed a significant decrease in antioxidant capacity (*p* < 0.05), with reductions of 39% (from 68.70 to 41.83%) and 87% (from 70.21 to 9.13%), respectively, between the gastric and intestinal phases. The pulp, in turn, did not present a statistically significant difference between the respective phases. This phenomenon is related to the fact that free radicals, unstable molecules, can induce damage to various cellular structures, including lipids, proteins, and DNA, facilitating the process of premature aging and the development of multiple pathologies.^75^ Therefore, antioxidant compounds that neutralize these reactive species play a crucial role in maintaining redox balance, preventing the emergence of chronic oxidative stress. However, for these compounds to exert their beneficial effects, it is essential to ensure their stability and activity during the in vitro digestive process to ensure their adequate intestinal absorption and subsequent transport to the target tissues.^79^

**Figure 1:**
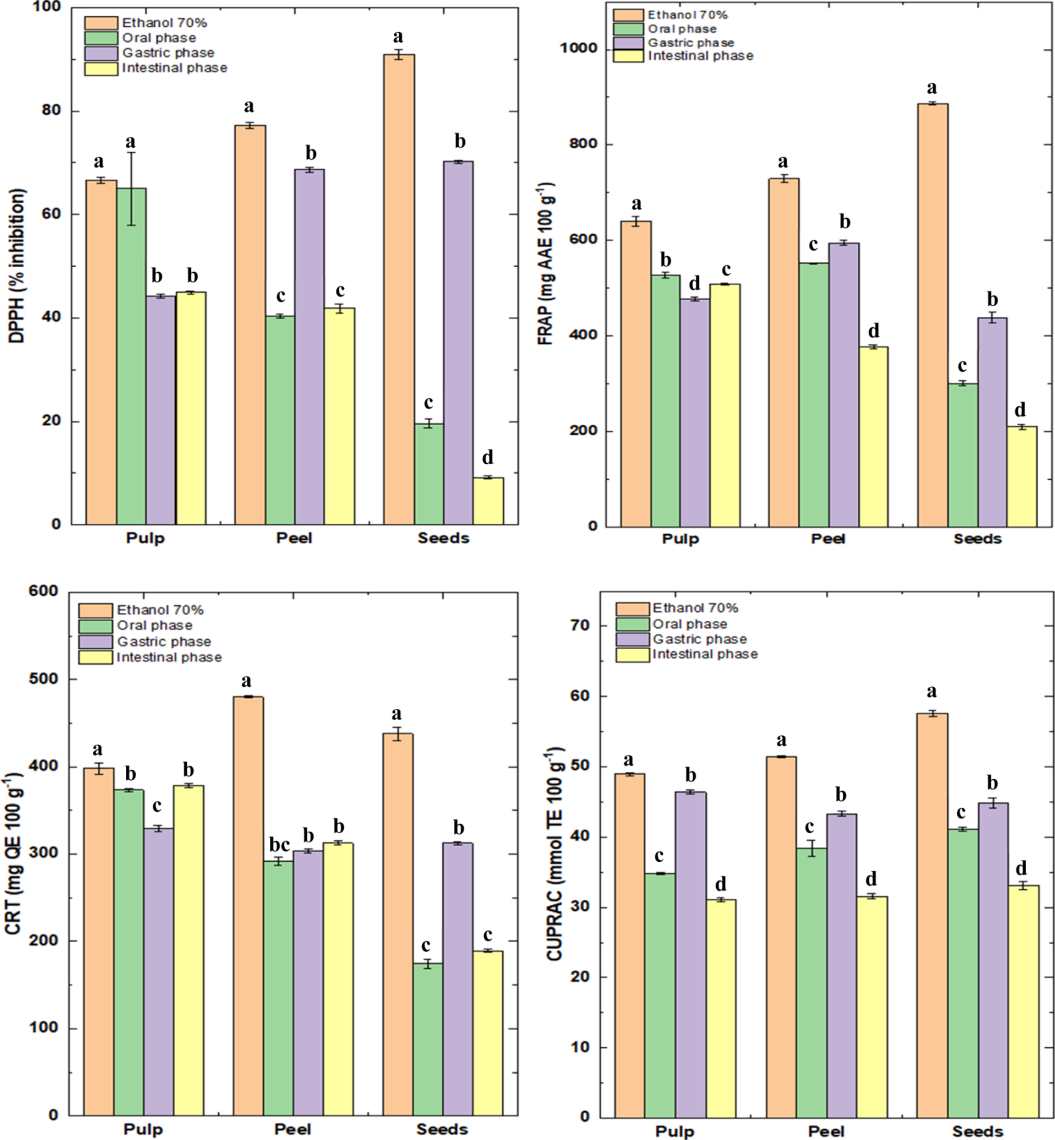
Antioxidant potential of the pulp, peels, and seeds of yellow puçá before, during, and after simulation of in vitro gastrointestinal digestion.

The CRT and FRAP analyses presented in Figure 2 show a significant reduction in antioxidant activity throughout the simulated gastrointestinal digestion phases, particularly in the intestinal phase, for all yellow puçá fractions (pulp, peel, and seed). While the ethanolic extracts presented the highest values for both CRT (397.90–479.90 mg QE 100 g^–1^) and FRAP (640.96–887.12 mg AAE 100 g^–1^), reflecting the high extraction of phenolic compounds under ideal solvent conditions. However, a significant decrease in these values was observed in the oral and gastric phases, and even more pronounced in the intestinal phase, suggesting degradation or structural transformation of the bioactive compounds due to changes in pH and enzymatic action. Notably, the husk showed greater relative stability in CRT between the gastric and intestinal phases (from 303.65 to 312.53 mg QE 100 g^–1^), while the seed showed greater loss of antioxidant potential by the FRAP protocol (from 887.12 undigested extract to 209.87 mg AAE 100 g^–1^ intestinal phase), indicating specific sensitivity of its phenolic matrix. These results reinforce the importance of considering the real bioaccessibility of antioxidants, and not only their total levels, when evaluating the functional potential of foods.

**Figure 2:**
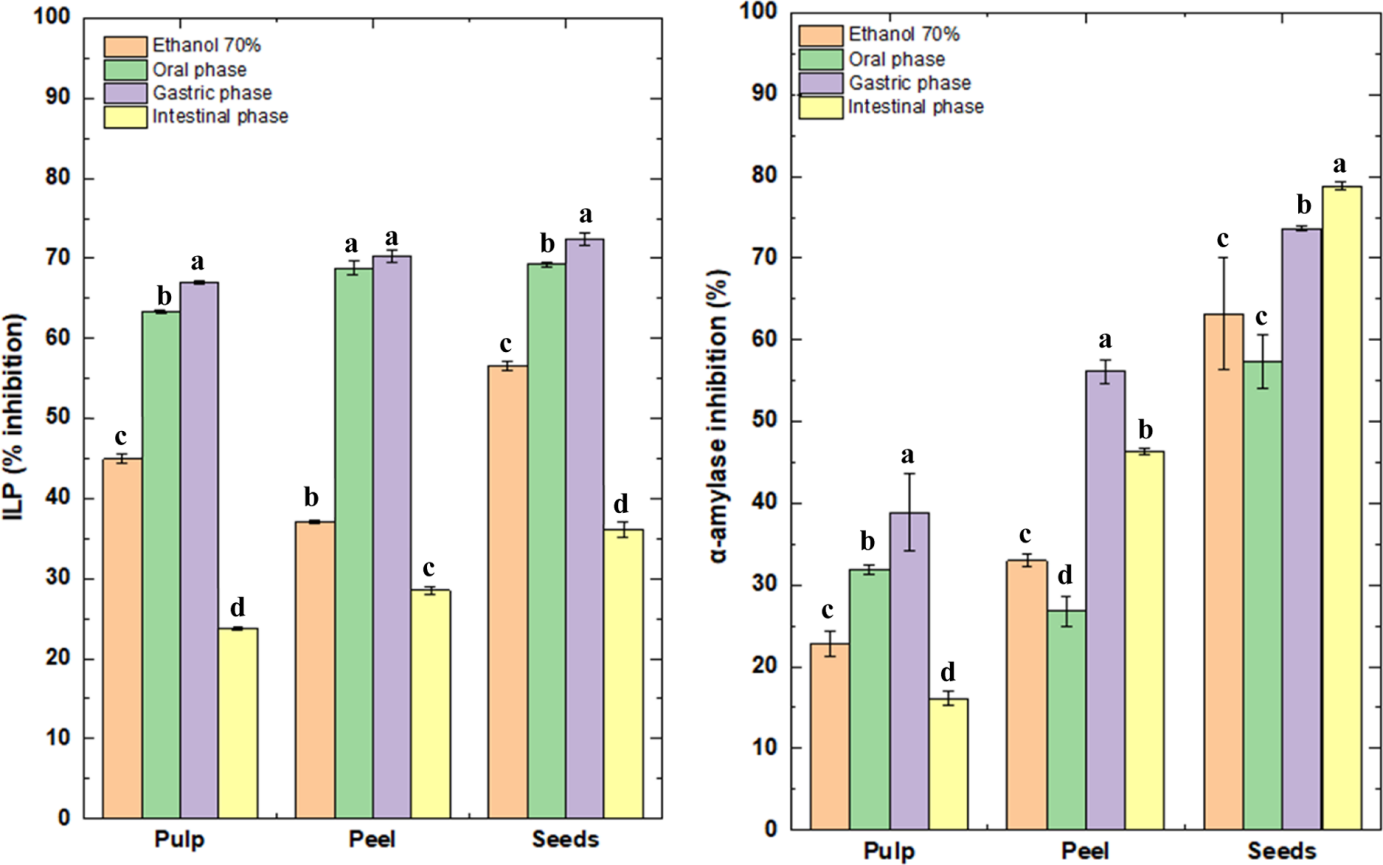
Biological properties of yellow puçá pulp, peels, and seeds before, during, and after simulation of in vitro gastrointestinal digestion.

The CUPRAC method showed the best results among the other antioxidant assays evaluated and showed the smallest reductions between the undigested sample and the intestinal phase (*p* < 0.05), with reductions of 36.50% (from 48.92 to 31.06 mmol TE 100 g^–1^), 38.61% (from 51.43 to 31.57 mmol TE 100 g^–1^) and 42.56% (from 57.60 to 33.08 mmol TE 100 g^–1^), for pulp, peels and seeds, respectively. These reductions may be related to pH changes in the in vitro digestion simulation phases.^80^ This suggests that even after the entire gastrointestinal digestion process, the antioxidant action responsible for the beneficial health effects of consuming these fruits, which are rich in antioxidants, may still be maintained.^81^ The data also indicate that most of the digested fractions presented favorable results, corroborating the results presented in the phenolic profile.

The decrease in antioxidant potential at the end of the simulated digestion process may suggest that the compounds are unstable against changes in pH and enzymatic activity. Antioxidant compounds demonstrate greater reactivity in acidic pH conditions, typical of gastric digestion, and less reactivity at pH close to neutrality, as occurs in intestinal digestion.^82^ In this context, during the digestive process, the biotransformation of the original compounds into others with lower antioxidant capacity may occur, or even the formation of new metabolites with preserved or potentially more effective antioxidant properties.^83,84^ However, it is possible to observe that most of the fractions, after digestion, presented satisfactory results in terms of antioxidant capacity, indicating that the antioxidant metabolites maintained their protective potential. This protective potential thus contributes to reducing inflammation and chronic diseases, such as cardiovascular diseases, diabetes, and neurodegenerative diseases.

### 3.8 In Vitro Biological Properties

The in vitro biological properties of the pulp, peels, and seeds of yellow puçá are shown in Figure 2. The highest inhibition percentages were recorded in the samples from the gastric phase, both for lipid peroxidation inhibition (ILP) and for α-amylase inhibition, in all fruit fractions. In the ILP analysis, a significant increase was observed between the undigested samples and those from the gastric phase (*p* < 0.05). The pulp showed an increase of 45.74% (from 44.95% to 66.97% inhibition), the peels showed an increase of 89.37% (from 37.09% to 70.24%), and the seeds showed an increase of 15.78% (from 56.56% to 72.34%). Despite the promising results observed in the gastric phase, the last stage of the process, corresponding to the intestinal phase, showed significant reductions in the lipid peroxidation inhibition values (*p* < 0.05), with levels lower than those of the undigested samples, indicating substantial variations between the different matrices and the phases of the in vitro digestive process. This decrease in the inhibition potential throughout the digestive phases is corroborated by studies by Chen et al.,^85^ which suggest that digestion can negatively impact the antioxidant potential, mainly due to the reduction in the content of phenolic compounds, which can be transformed into altered structural forms with distinct chemical properties. Additional factors, such as pH variations and particle size during the digestive process, can also contribute to the decrease in the inhibition potential observed in the different phases of digestion.

Figure 2 shows the results of α-amylase enzyme inhibition before, during, and after the simulated in vitro gastrointestinal digestion process. Notably, the gastric phase showed the highest inhibition levels (38.83 and 56.40% inhibition) for pulp and peels and decreased significantly in the intestinal phase with reductions of 58.48% (from 38.83 to 16.12%) and peels with reductions of 17.47% (from 56.10 to 46.30%) (*p* < 0.05). These results are by Burgos-Edwards et al.,^86^ where the authors reported that the α-amylase inhibitory activities of two wild Chilean gooseberries (*Ribes magellanicum* and *Ribes punctatum*) decreased after gastric digestion. These differences may be due to the different compositions of the phytochemical components and their affinity with the respective phases. On the other hand, the highest percentage of seed inhibition is related to the intestinal phase (78.84%), with an increase of 7.08% between the gastric and intestinal phases, corroborating the findings of He et al.^87^ when analyzing 22 juices from native and traditional fruits, noticed that after the gastric phase, some juices presented a higher percentage of inhibition compared to the other stages of the in vitro digestion process. These results suggest that the digestive process modulates the inhibitory activity of α-amylase differently, depending on the digestion phase and the phytochemical composition of the fractions analyzed.

## 4 Conclusions

The findings of this study highlight the nutritional and functional potential of the different fractions of yellow puçá. Peels and seeds were rich in lipids, proteins, and dietary fibers, while the pulp exhibited high levels of pectin, fructose, and malic acid—compounds of interest for food and health-related applications. The oils extracted showed a predominance of palmitic acid, indicating a saturated profile with potential thermal stability, making them suitable for industrial uses, particularly in frying processes. All fractions demonstrated satisfactory levels of essential minerals, amino acids, and triacylglycerols. Gallic acid was the main phenolic compound identified in both fractions, reinforcing their value for pharmaceutical and cosmetic applications due to its well-documented antioxidant and bioactive properties. However, in vitro gastrointestinal digestion led to a marked reduction in phenolic content, antioxidant activity, and related biofunctions, especially during the intestinal phase. These results suggest that digestive conditions can significantly affect the stability and bioaccessibility of phenolic compounds, pointing to the occurrence of degradation or transformation mechanisms that warrant further investigation. Overall, this study underscores the complexity of the interactions between food bioactives and the human digestive system and emphasizes the importance of deepening our understanding of these processes to better harness the health benefits and industrial applications of underutilized native fruits such as yellow puçá.

## Data Availability

The datasets generated during and analyzed during the current study are available from the corresponding author upon reasonable request.
